# *Haemophilus influenzae* type b serology in childhood leukaemia: A case–control study

**DOI:** 10.1054/bjoc.2001.1903

**Published:** 2001-08

**Authors:** F D Groves, D Sinha, H Kayhty, J J Goedert, P H Levine

**Affiliations:** Department of Biometry and Epidemiology, Medical University of South Carolina, Charleston, South Carolina, USA; Biostatistics Branch, National Cancer Institute, Rockville, Maryland, USA; Laboratory of Vaccine Immunology, National Public Health Institute, Helsinki, Finland; Viral Epidemiology Branch, National Cancer Institute, Rockville, Maryland, USA

**Keywords:** leukaemia, child, serology, *Haemophilus influenzae* type b, case–control study

## Abstract

Antibody to *Haemophilus influenzae* type b (Hib) polysaccharide (PRP) was measured in 42 children with acute lymphoblastic leukaemia (ALL) and 42 non-leukaemic hospital controls. Modelling anti-PRP concentrations as a function of age revealed that the slopes of the trend lines differed significantly between cases and controls (*P* = 0.05); anti-PRP concentrations were lower among younger cases, and higher among older cases, than among controls of the same ages. © 2001 Cancer Research Campaign http://www.bjcancer.com


British Journal of Cancer (2001) 85(3), 337–340
© 2001 Cancer Research Campaign
doi: 10.1054/ bjoc.2000.1903, available online at http://www.idealibrary.com on IDEAL®

http://www.bjcancer.com

Short Communication

### *Haemophilus influenzae* type b serology in childhood leukaemia: A case–control study

**FD Groves[1,2], D Sinha[1], H Kayhty[3], JJ Goedert[4] and PH Levine[4]**

[1]Department of Biometry and Epidemiology, Medical University of South Carolina, Charleston, South Carolina, USA; [2]Biostatistics Branch, National Cancer Institute, Rockville, Maryland, USA; [3]Laboratory of Vaccine Immunology, National Public Health Institute, Helsinki, Finland; [4]Viral Epidemiology Branch, National Cancer Institute, Rockville, Maryland, USA

**Summary** Antibody to *Haemophilus influenzae* type b (Hib) polysaccharide (PRP) was measured in 42 children with acute lymphoblastic leukaemia (ALL) and 42 non-leukaemic hospital controls. Modelling anti-PRP concentrations as a function of age revealed that the slopes of the trend lines differed significantly between cases and controls ($P = 0.05$); anti-PRP concentrations were lower among younger cases, and higher among older cases, than among controls of the same ages. © 2001 Cancer Research Campaign http://www.bjcancer.com

**Keywords**: leukaemia; child; serology; *Haemophilus influenzae* type b; case–control study

A recent US case–control found an inverse association between exposure to Hib conjugate vaccine and subsequent risk of childhood ALL (Groves et al, 1999). These findings were corroborated by reanalysis of data from a clinical trial of conjugate Hib vaccine in Finland in the 1980s (Eskola et al, 1990), which suggested that administration of the vaccine before the first birthday, but not after, was associated with a reduced risk of childhood ALL (Auvinen et al, 2000). To further evaluate the possible relationship between Hib antigen exposure and risk of ALL, the present study compared anti-PRP concentrations in banked sera from Canadian childhood ALL cases and matched controls.

### MATERIAL AND METHODS

### Serum selection

Sera from 42 ALL patients were obtained from a repository of specimens that had been collected between 1966 and 1970 from the Hospital for Sick Children in Toronto and the Montreal Children's Hospital. Their median age was 5 years (range: 1 to 17 years), with 23 boys and 19 girls. Sera also were collected from 42 contemporaneous age- and sex-matched non-leukaemic hospital controls. All 42 case–control pairs were matched on hospital and age (± 4 years); 39 pairs also were matched on sex. The controls included 12 children with various forms of anaemia; 7 children suffering from complications of treatment; 4 children with coagulation deficits; 4 children with rheumatic fever or its sequelae; 3 children described only by their symptoms; 2 children with asthma; 2 children with congenital disorders of lipid metabolism; and 7 children with various other diagnoses (see table).

Reagent sera with high (36.6 μg ml$^{-1}$) and low (0.34 μg ml$^{-1}$) anti-Hib antibody concentrations were provided by Dr. Carl Frasch (United States Center for Biologics Evaluation and Research, Food and Drug Administration (CBER/FDA)). An intermediate concentration of standard sera was prepared by mixing the high- and low-concentration standard sera in a 1:20 ratio, for a final concentration of 2.15 μg ml$^{-1}$. In identical vials, the 84 case-control sera were interspersed with replicates of the three reagent standard sera, arranged in random order, and tested under code.

### Laboratory methods

Hib serology was performed by the Laboratory of Vaccine Immunology of the National Public Health Institute in Helsinki, Finland as described (Kayhty et al, 1987; Phipps et al, 1990). Briefly, wells of EIA plates (Costar 3591, Costar Corporation, Cambridge MA, USA) were coated with 100 μl HbOHA antigen (lot 15D, Dr Moon Nahm, Rochester University, Rochester, NY, USA); 1 μg ml$^{-1}$ in phosphate buffered saline (PBS). Serum samples were diluted into PBS containing 10% of fetal calf serum (KC Biologicals Inc, Lenexa, Kansas); and 100 μl was added into each well. The detecting antibody was horseradish peroxidase conjugated rabbit anti-human immunoglobulin preparation (P212, Dakopatts, Denmark). After incubation, the substrate solution (1.1 M Na acetate in distilled water with 0.6% TMB and 30% $H_2O_2$) was added and the reaction was stopped with 2 M $H_2SO_4$ after incubation. The optical density (OD) was read with the Titertek Multiskan spectrophotometer. A reference serum (lot 1983, received from Dr Carl Frasch, CBER/FDA, Bethesda, Md) containing 70 μg of anti-PRP ml$^{-1}$ was run on each plate. The results were given as μg of anti-PRP ml$^{-1}$. The lowest detectable concentration of anti-PRP was 0.10 μg ml$^{-1}$.

### Statistical methods

All 42 matched pairs were analysed by conditional logistic regression using SAS PROC PHREG. The 22 cases aged ≤ 5 years and their matched controls were analysed separately, as were the 20 cases aged > five years and their matched pairs. Then the matching was broken, and unconditional logistic regression (SAS PROC

*Received 28 February 2001*
*Revised 24 April 2001*
*Accepted 25 April 2001*

*Correspondence to:* FD Groves

Diagnoses of non-leukaemic hospital control children

| | | | | | |
|---|---|---|---|---|---|
| Anaemias | | | | | 12 |
| | hereditary haemolytic | | | 8 | |
| | | Mediterranean | 6 | | |
| | | due to haemoglobinopathies | 2 | | |
| | iron deficiency | | | 1 | |
| | aplastic | | | 2 | |
| | unspecified | | | 1 | |
| Complications of... | | | | | 7 |
| | ...medical care | | | 6 | |
| | ...surgical procedures | | | 1 | |
| Coagulation deficits | | | | | 4 |
| | haemophilia | | | 3 | |
| | other | | | 1 | |
| Rheumatic fever and its sequelae | | | | | 4 |
| | Rheumatic fever, without mention of heart involvement | | | 2 | |
| | Other heart diseases, specified as rheumatic | | | 1 | |
| | [Sydenham's] chorea, without mention of heart involvement | | | 1 | |
| Other diseases of blood | | | | | 1 |
| Multiple myeloma | | | | | 1 |
| Asthma | | | | | 2 |
| Congenital disorders of lipid metabolism | | | | | 1 |
| Epilepsy | | | | | 1 |
| Psoriasis and similar disorders | | | | | 1 |
| Spina bifida without mention of hydrocephalus | | | | | 1 |
| Cerebral spastic infantile paralysis | | | | | 1 |
| Anomaly of the lower limb | | | | | 1 |
| Symptoms referable to the... | | | | | 3 |
| | ...abdomen | | | 1 | |
| | ...nervous system and senses | | | 1 | |
| | ...cardiovascular and lymphatic systems | | | 1 | |

LOGISTIC) was used to analyse all 84 subjects together, after which the 39 subjects aged ≤ 5 years were analysed separately, as were the 45 subjects aged > 5 years. Throughout the analysis, the odds ratio, its 95% confidence interval, and its associated two-sided *P* value, were calculated, with the low Hib antibody concentration being the referent group.

Antibody concentration then was expressed as a function of age for the cases and controls separately, and the slopes of the regression lines were compared. A random-effects model was used to allow for the effects of matching. When anti-PRP was undetectable, a concentration of 0.05 μg ml$^{-1}$ (half the lower limit of detection) was imputed for purposes of the regression analysis. For the ith pair, the conditional regression equation was given by $Y_{ij} = \alpha_j + \beta_j X_{ij} + e_{ij}$, where j = 1 for the case and j = 2 for the control, $Y_{ij}$ was the antibody concentration and $X_{ij}$ was the age for the (i,j)th child, and $e_{ij}$ was the corresponding error term. To incorporate the random-effect of the ith pair, we assumed that $e_{i1}$ and $e_{i2}$ followed a bivariate-normal distribution with mean = 0 and unknown dispersion matrix Σ. The analysis was performed using the procedures for repeated measurements data in SAS PROC MIXED.

### RESULTS

The ages in years of cases (median = 5; mean = 6.29; standard error = 0.59) and controls (median = 6; mean = 7.26; standard error = 0.68) were similar. There were 23 male cases and 29 male controls. Anti-PRP concentrations in the high-, intermediate-, and low-concentration standard sera ranged from 25.42 to 45.50 μg ml$^{-1}$, from 1.53 to 3.60 μg ml$^{-1}$, and from 0.28 to 0.84 μg ml$^{-1}$, respectively. Among the controls, anti-PRP was undetectable in 5 subjects but concentrations ranged up to 23.30 μg ml$^{-1}$, with a

*© 2001 Cancer Research Campaign*

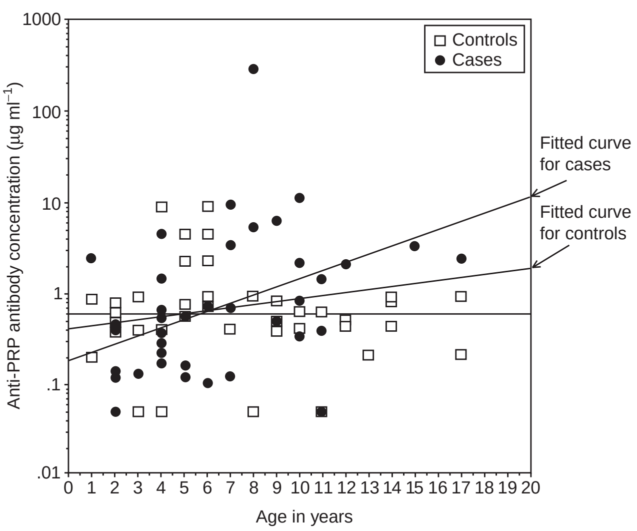

**Figure 1** Anti-PRP antibody concentration as a function of age, with fitted curves that differed significantly (*P* = 0.05) between cases and controls median concentration of 0.60 μg ml$^{-1}$. Among the cases, anti-PRP was undetectable in 3 subjects but concentrations ranged up to 286.25 μg ml$^{-1}$, with a median concentration of 0.54 μg ml$^{-1}$.

Conditional logistic regression showed no association of anti-PRP with risk of ALL (odds ratio = 0.80, $P = 0.64$) when data from children of all ages were combined. For the 22 pairs in which the case's age at diagnosis was ≤ 5 years, an anti-PRP concentration > 0.60 μg ml$^{-1}$ was inversely associated with ALL (odds ratio = 0.29, $P = 0.12$). Among the 20 matched pairs in which the case was diagnosed at age ≥ 6 years, however, a high anti-PRP concentration was associated with an increased risk of ALL (odds ratio = 2.00, $P = 0.33$).

Unconditional logistic regression likewise revealed no association of anti-PRP with risk of ALL (odds ratio = 0.83, $P = 0.66$) when the unmatched data were analyzed for children of all ages. Among 39 subjects aged ≤ 5 years, an anti-PRP concentration > 0.60 μg ml$^{-1}$ was inversely associated with risk of ALL (odds ratio = 0.25, $P = 0.06$). The opposite was true among the 45 subjects over the age of 5 years, an anti-PRP concentration > 0.60 μg ml$^{-1}$ was associated with an increased risk of ALL (odds ratio = 2.77, $P = 0.12$).

When antibody concentration was expressed as a function of the ages of the cases and controls separately, the 2 regression lines had distinctly different slopes ($P = 0.05$). Inspection of Figure 1 reveals that younger cases generally had lower antibody concentrations than younger controls, while the opposite was true for the older subjects. This finding was not altered by exclusion of one subject with an extremely high anti-PRP concentration (286.25 μg ml$^{-1}$, data not shown).

### DISCUSSION

We found a lower risk of ALL among Canadian preschool children with higher concentrations of anti-PRP. Among older children, however, higher anti-PRP concentrations were associated with an increased risk of ALL. As these sera were collected before Hib vaccines were developed, the observed antibody concentrations cannot be attributed to Hib vaccination, but must reflect actual exposure to PRP from infection with Hib or cross-reacting organisms. These findings suggest that early exposure to such infectious agents may protect against childhood ALL, while delayed exposure may increase the risk of ALL. Some limited evidence has been provided for an effect of common infections on the risk of childhood leukaemia (Greaves, 1997). Greaves has postulated that childhood ALL is initiated in utero, but that modulation of the immune system by common antigenic exposures during early infancy can somehow suppress the expansion of the preleukaemic cell population, whereas a delay in the antigenic exposures to later ages may drive the preleukaemic clone to proliferate, increasing the risk of subsequent childhood ALL (Greaves, 1999).

Previous epidemiologic studies of Hib vaccine and childhood ALL have lent support to the Greaves hypothesis. A large ($n$ = 439) matched case–control study in the United States found a substantially lower risk of ALL (odds ratio (OR), 0.55; 95% confidence interval (CI), 0.35–0.87) among children who had been vaccinated against Hib during the era when conjugate vaccine was predominant (Groves et al, 1999). Because Hib vaccination was only one of several types of early childhood vaccinations studied and there was no clear a priori hypothesis or obvious biological mechanism, it is unclear if the possible protective effect might be due to the vaccination itself, to avoidance of infection, or to chance, bias, or confounding by an unknown variable. In a controlled clinical trial conducted in Finland (Auvinen et al, 2000), there was a non-significant decreased risk (RR = 0.72; 95% CI = 0.46–1.13) of childhood leukaemia among subjects who received multiple doses of Hib conjugate vaccine in the first year of life, compared with those whose only dose was delayed until the second birthday.

The mechanism by which early antigenic exposures might alter the risk of subsequent childhood ALL is unclear. Many childhood ALL cases arise from a monoclonal expansion of cells with a characteristic TEL-AML1 fusion gene (Wiemels et al, 1999). Twin studies have shown that this clonal cell population may occur even in the non-leukaemic identical twins of childhood leukaemia cases. Remarkably, the concordance rate for leukaemia in identical twins is only about 5% (Greaves, 1993). Thus, the TEL-AML1 fusion gene is not sufficient for ALL, since most clones do not evolve into leukaemia. Clearly, postnatal exposures play an important role in promoting the development of leukaemia from the preleukemic clone.

One weakness of the present study is that the sera were obtained from cases only after ALL was diagnosed; thus, the anti-PRP concentrations might have been altered by the disease process itself. Unvaccinated leukaemic children aged 2 to 6 years at St Jude Children's Research Hospital had lower anti-PRP concentrations than nonleukaemic children. Nonetheless, most of the leukaemic children who had received chemotherapy for less than 12 months were still able to mount an antibody response to a conjugate Hib vaccine, suggesting that they had not been exposed to the PRP antigen previously (Feldman et al, 1990). Furthermore, the older ALL cases in our study had elevated, rather than depressed, levels of anti-PRP, again suggesting that the disease process does not obliterate the immune response to PRP. One strength of our study is that our sera were obtained in an era when there were no Hib vaccines; thus, the observed anti-PRP concentrations must reflect actual infection with Hib or cross-reacting organisms. The present study is consistent with the Greaves hypothesis that early antigenic exposures protect against childhood ALL, while delayed exposure increases the risk.

*© 2001 Cancer Research Campaign*

### ACKNOWLEDGEMENTS

The authors are grateful for the assistance of Ms Kathi Shea and Ms Carla Hanson (NO2-CP-71001 to BBI-Biotech, Inc.) and Ms Violet Devariakkam (N02-CP-91027 to Research Triangle Institute) for specimen and study management, Ms Leena Saarinen (N01-CO-5600 to Scientific Applications International Corporation) for the antibody analyses, and Mr Aaron Adelman for preparation of the figure.

### REFERENCES

Auvinen A, Hakulinen T and Groves FD (2000) *Haemophilus influenzae* type b vaccination and risk of childhood leukaemia in a vaccine trial in Finland. *Br J Cancer* **83**: 956–958

Eskola J, Käyhty H, Takala AK, Peltola H, Rönnberg P-R, Kela E, et al (1990) A randomized, prospective field trial of a conjugate vaccine in the protection of infants and young children against invasive *Haemophilus influenzae* type b disease. *N Engl J Med* **323**: 1381–1387

Feldman S, Gigliotti F, Shenep JL, Roberson PK and Lott L (1990) Risk of *Haemophilus influenzae* type b disease in children with cancer and response of immunocompromised leukemic children to a conjugate vaccine. *J Infect Dis* **161**: 926–931

Greaves M (1993) A natural history for paediatric acute leukaemia. *Blood* **82**: 1043–1051

Greaves MF (1997) Aetiology of acute leukaemia. *Lancet* **349**: 344–349

Greaves MF (1999) Molecular genetics, natural history, and the demise of childhood leukaemia. *Eur J Cancer* **35**: 173–185

Groves FD, Gridley G, Wacholder S, Shu X-O, Robison LL, Neglia J and Linet MS (1999) Infant vaccinations and risk of childhood acute lymphoblastic leukaemia in the United States. *Br J Cancer* **81**: 175–178

Käyhty H, Eskola J, Peltola H, Stout M, Samuelson JS and Gordon LK (1987) Immunogenicity in infants of a vaccine composed of *Haemophilus influenzae* type b capsular polysaccharide mixed with DPT or conjugated to diphtheria toxoid. *J Infect Dis* **155**: 100–106

Phipps DC, West J, Eby R, Koster M, Madore DV and Quataert SA (1990) An ELISA employing a *Haemophilus influenzae* type b oligosaccharide-human serum albumin conjugate correlates with the radioantigen binding assay. *J Immunol Methods* **135**: 121–8

Wiemels JL, Cazzaniga G, Daniotti M, Eden OB, Addison GM, Masera G, Saha V, Biondi A and Greaves MF (1999) *Lancet* **354**: 1499–1503

*© 2001 Cancer Research Campaign*

